# The potential use of scout views as an adjunct tool in CT-guided intervention

**DOI:** 10.1038/s41598-025-22040-z

**Published:** 2025-10-01

**Authors:** Dominic Conroy, Thomas Werncke, Stephan Waldeck, Frank Wacker, Christian von Falck

**Affiliations:** 1 Institute for Diagnostic and Interventional Radiology, Hannover Medical School, Carl-Neuberg-Strasse 1, 30625 Hannover, Germany; 2 Department of Radiology and Neuroradiology, Bundeswehr Central Hospital, Rübenacher Strasse 170, 56072 Koblenz, Germany

**Keywords:** Medical research, Imaging, X-ray tomography

## Abstract

The objective of this study was to evaluate the use of scout views as an adjunct tool in CT-guided intervention. Therefore, different devices used for interventional procedures were assigned to three different groups and placed onto an Alderson phantom. Different body diameters were simulated by adding PMMA/aluminum plates. Multiple scout views (ap) were acquired using a standard CT-scanner with different scan parameters. The visibility of the devices was semi-quantitatively scored by three readers. The interrater reliability was calculated using the intraclass correlation coefficient (ICC). The detectability of the 11 different interventional devices was calculated depending on tube potential, tube current–time product, and the thickness of the additional tissue equivalent. Statistical significance was evaluated using Wilcoxon signed-rank test (with Pratt modification) and the Mann–Whitney-U test, respectively. The ICC over all measurements for the three readers as well as the ICC for the three different devices groups was regarded excellent (> 0.9). The device detectability was significantly different for the different additional tissue thicknesses as well as for the three device groups. Material detectability over all readers improved with higher doses. Defining a median visibility of 3 (“good”) or 4 (“very good”) as a cut-off value for clinical routine use, we consider a combination of ≥ 80kVp / ≥ 60 mAs or ≥ 90 kVp / ≥ 40 mAs as reasonable scan parameters in normal and overweight patients in our CT setup. In conclusion, CT scout views have a potential use as an additional tool in CT-guided-intervention by enabling fluoroscopy-like planar imaging that may help to visualize dynamic events such as contrast injection or guidewire manipulation.

## Introduction

CT-guidance is an indispensable tool for percutaneous image-guided interventions in radiology. It is used in a broad variety of minimally invasive procedures such as biopsies of solid structures, drainage of fluid collections, placement of catheters (e.g. nephrostomy, suprapubic catheter) as well as local-ablative therapies such as radiofrequency ablation (RFA), cryoablation, microwave ablation (MWA) or electrochemotherapy (ECT)^1^. CT creates distortion- and (almost) artifact-free cross-sectional images of all parts of the body and therefore allows precise needle placement even in challenging anatomical situations. The standard procedure in interventional CT is usually an iterative approach: After diagnostic CT images are obtained, a trajectory is planned based on these images. Then the needle is incrementally advanced towards the lesion while repetitive thin-section low-dose CT scans are acquired to compare the actual needle position within the patient to the planned trajectory^2^. Although 2D real-time imaging with CT is available (CT fluoroscopy), it comes with the burden of increased radiation exposure to the patient as well as to the interventionalist^3–5^.

However, in certain CT-guided interventions, real-time fluoroscopic imaging might facilitate the procedure by visualizing dynamic events such as the injection of contrast agents or manipulation with guide wires^6^. Some groups and manufacturers have addressed this need by placing a C-arm in front of the CT^7–9^. However, every CT scanner has the intrinsic ability to generate fluoroscopic-like planar images. Therefore, the aim of this study is to systematically evaluate the possible use of scout views in CT-guided interventions.

## Methods

### Phantom

An antropomorphic Alderson phantom (Atom Dosimetry Verification Phantom, CIRS Inc., Norfolk, VA, USA) with an abdominal cross-sectional diameter of 20 × 30 cm was placed onto 1, 4 and 8 composite plates, respectively. Each plate consisted of 20 mm PMMA and 2 mm aluminum (representing 25 mm of tissue equivalent material), resulting in an additional tissue thickness of 25 mm, 100 mm and 200 mm, respectively. According to O’Neill et al. these phantom sizes correspond to patients with a body mass index of approximately 22 (normal weight), 27 (overweight) and 33 (adiposity stage 1), respectively^10^.

### Materials

**Fig. 1:**
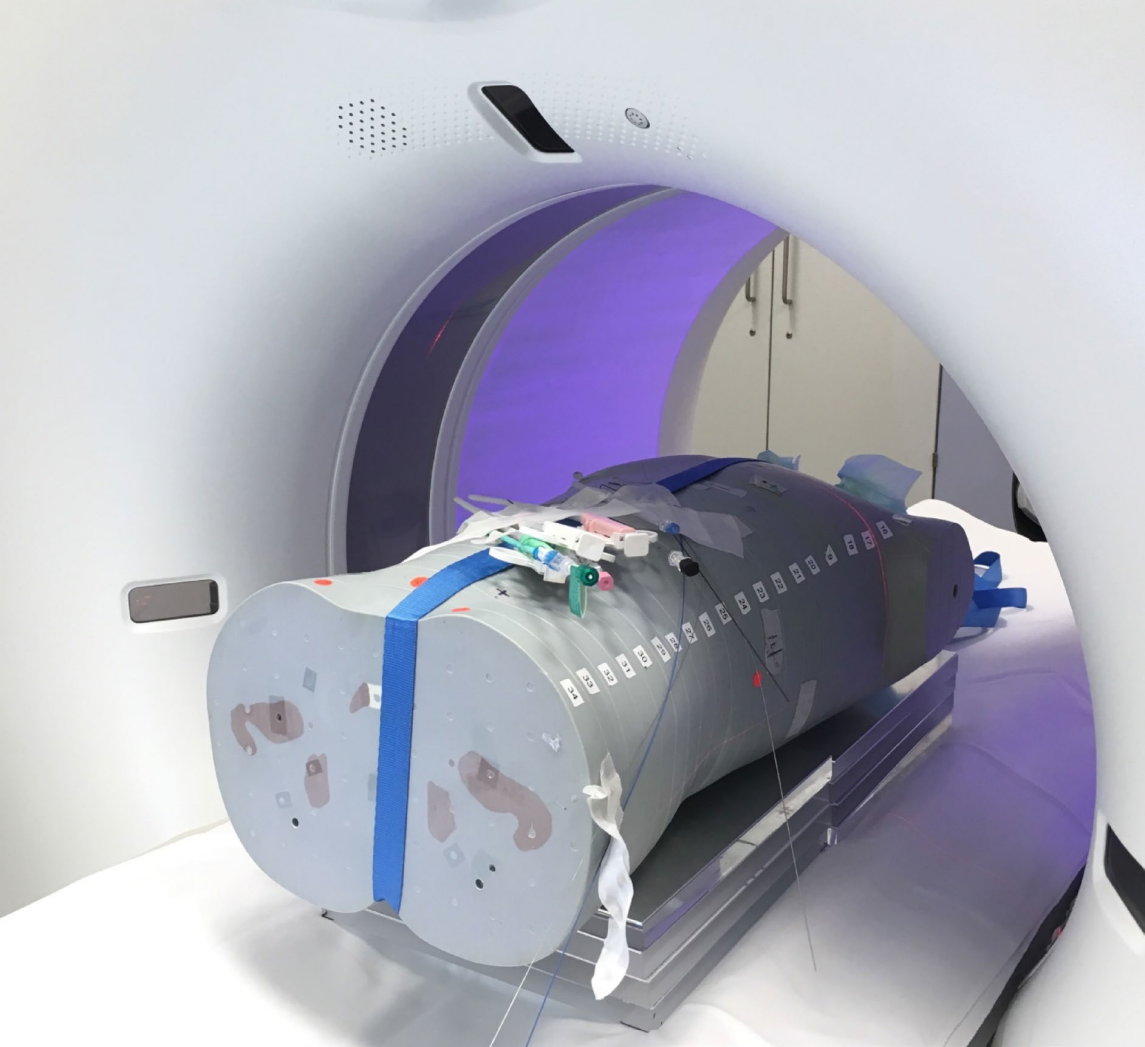
Photography of the experimental setting. The anthropomorphic phantom ist placed on the CT table. The different materials are placed onto the phantom. The composite plates are placed between the scanner table and the phantom.

Different standard interventional devices used for interventional procedures (n = 11) were placed onto the Alderson phantom: a 5F vascular access sheath with dilator (Cordis Avanti, Cordis Germany GmbH, Deutschland), single action biopsy devices of different diameters (14G, 16G, 18G; Manan Medical Products, USA), chiba needles of different diameters (17G, 22G, 25G; Cook Medical Deutschland GmbH, Germany) and different guide wires (0.035″ TERUMO Glidewire, TERUMO Deutschland GmbH, Germany; 0.035″ TERUMO Radiofocus Sheath guidewire; 0.035″ Boston Amplatz Superstiff, Boston Scientific Medizintechnik GmbH, Germany; 0.038″ Cordis Avanti Sheath Mini-wire). Based on the imaging characteristics and similarity in detectability during a standard fluoroscopic examination, the devices were assigned to three different groups that were used for further statistical analysis (see Table 1). Group 1 consisted of the 5F sheath, the three biopsy devices (14G, 16G, 18G) and the 17G chiba needle. Group 2 comprised the 22G and 25G chiba needles as well as the three different 0.035″ steel guidewires. The hydrophilic Glidewire was allocated to group 3. A photography of the experimental setting can be seen in Fig. 1.

**Table 1:** Overview of interventional devices used for this study.

Nr	Description	Group
1	5F AVANTI Vascular Access Sheath, Cordis	1
2	14G Single Action Biopsy Device, Manan Medical Products	1
3	16G Single Action Biopsy Device, Manan Medical Products	1
4	18G Single Action Biopsy Device, Manan Medical Products	1
5	17G Chiba Biopsy Needle, Cook Medical	1
6	22G Chiba Biopsy Needle, Cook Medical	2
7	25G Chiba Biopsy Needle, Cook Medical	2
8	0.035″ Guidewire, J-Type, Cook Medical	2
9	0.035″ Mini-Guidewire (Sheath kit), Terumo	2
10	0.035″ Amplatz Super Stiff, Boston Scientific	2
11	0.035″ Radifocus Glidewire (hydrophilic), Terumo	3

### Scan parameters

The phantom and the devices were placed at the isocenter of a clinical MDCT scanner (Somatom Force, Siemens Healthineers, Erlangen, Germany). Multiple scout views in the a.-p.-direction were acquired by systematically modifying tube potential (70 kVp, 80 kVp, 90 kVp, 100 kVp, 110 kVp, 120 kVp) and tube-current time product (20 mAs, 30 mAs, 40 mAs, 50 mAs, 60 mAs for 25 mm/100 mm/200 mm additional tissue thickness; 80mAs, 120 mAs, 160 mAs for 100 mm and 200 mm additional tissue thickness). The CTDI_vol_ and DLP for each scan were recorded.

For an indicative comparison of dose parameters of our approach with a standard fluoroscopic approach, the phantom was imaged using a standard angiography suite (Artis pheno, Siemens Healthineers, Erlangen, Germany). A standard clinical abdomen protocol (with reduced dose settings) was used. The collimation was adjusted to 10 cm (cranio-caudal direction) and a fluoroscopy loop of the phantom (with 25 mm/100 mm additional tissue thickness) was acquired. Dose parameters (dose area product) were extracted from the DICOM meta data. Effective dose was calculated using standard conversions factors for the abdomen according to AAPM^11^.

### Reader study

The resulting images were independently reviewed by three readers on a diagnostic DICOM-calibrated monitor (EIZO Radiforce, EIZO Europe GmbH, Germany) using a client–server PACS installation (Visage 7, Visage Imaging, Berlin, Germany). Image window settings could be modified at the discretion of the readers. The images were presented in random order and without any meta information with regard to the scan protocol. The detectability of the different devices was semi-quantitatively evaluated using a 5-point Likert scale (0—not visible, 1—poor, 2—moderate, 3—good, 4—very good). The readers were motivated to fully exploit the range of the Likert scale.

In order to establish reasonable scan parameters for practical use, a median visibility of 3 (“good”) or 4 (“very good”) was considered sufficient for clinical routine use by two interventional radiologists not involved in the study reading and therefore defined as the cut-off value for detectability in our study.

### Statistics

For statistical analysis, the open-source software package “R” (version 3.6.3, R Foundation for Statistical Computing, Vienna, Austria, https://www.R-project.org) and Microsoft Excel (Microsoft Corp., Redmond, WA, USA) were used. The interrater reliability was calculated using the intraclass correlation coefficient (ICC). The results for the three different readers were pooled and the detectability of the 11 different interventional devices was calculated depending on tube potential, tube current–time product, and the thickness of the additional tissue equivalent. Statistical significance was evaluated using Wilcoxon signed-rank test (with Pratt modification) and the Mann–Whitney-U test, respectively.

### Biopsy—proof-of-concept

In a second step, a custom phantom was made of gelatine and an iodinated contrast-agent (Imeron 400, Bracco Imaging, Konstanz, Germany) as a proof-of-concept for lesion targeting. The phantom was placed at the isocenter of the MDCT scanner and a diagnostic scan was acquired. The image dataset was reconstructed and the target was identified. The laser guidance of the scanner was used to target the slice of interest. From now on, the step-wise advancement of the needle was monitored using repetitive scout view acquisitions in an iterative approach. The success of the approach was proven using an intralesional placement of a guidewire as well as an injection of a small amount of contrast media.

## Results

### Reader study

#### Overall visibility

**Fig. 2:**
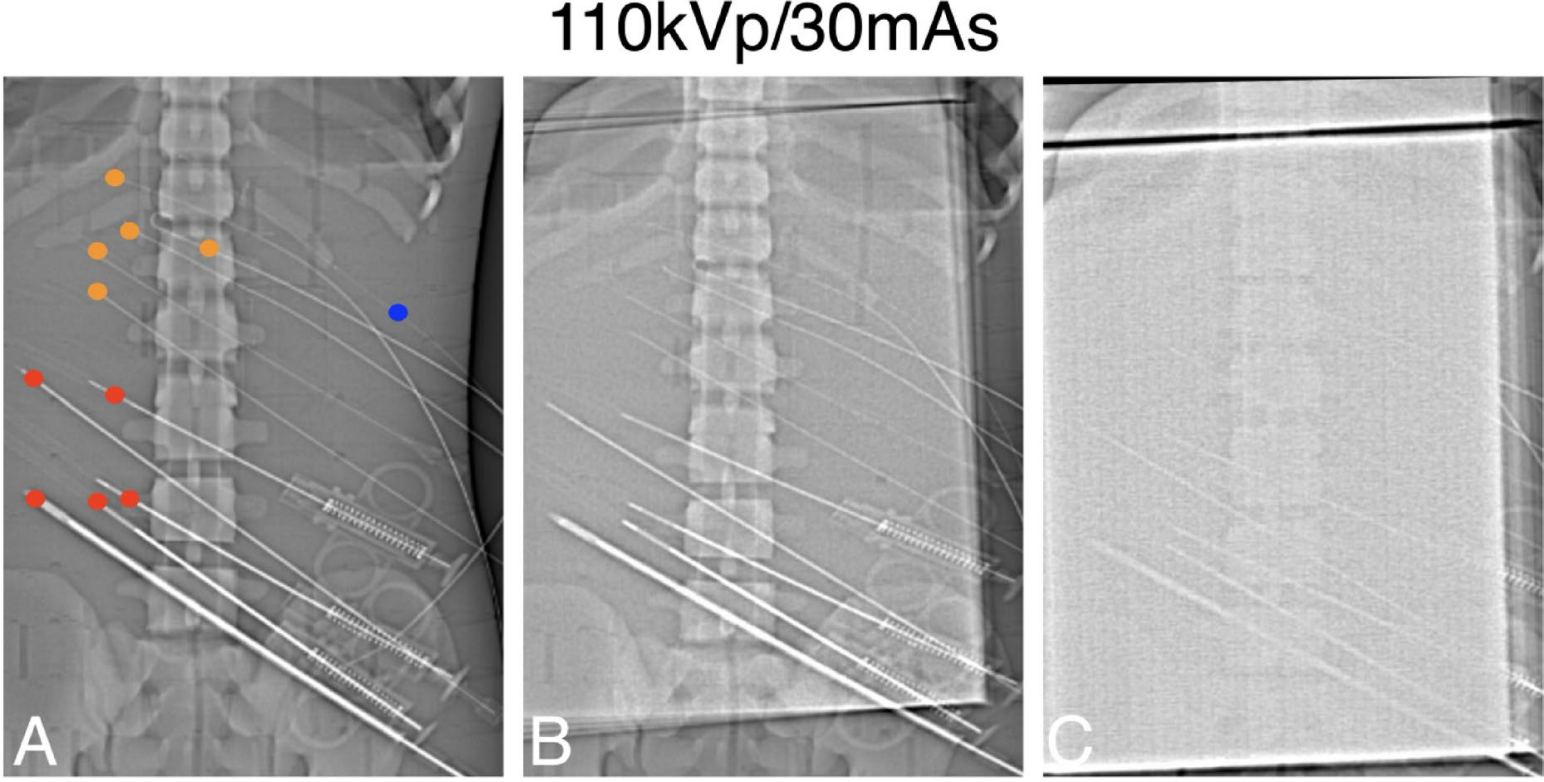
Visualization of different devices (red—group 1, orange—group 2, blue—group 3) with increasing tissue thickness (**A**: 25 mm, **B**: 100 mm, **C**: 200 mm).

**Fig. 3:**
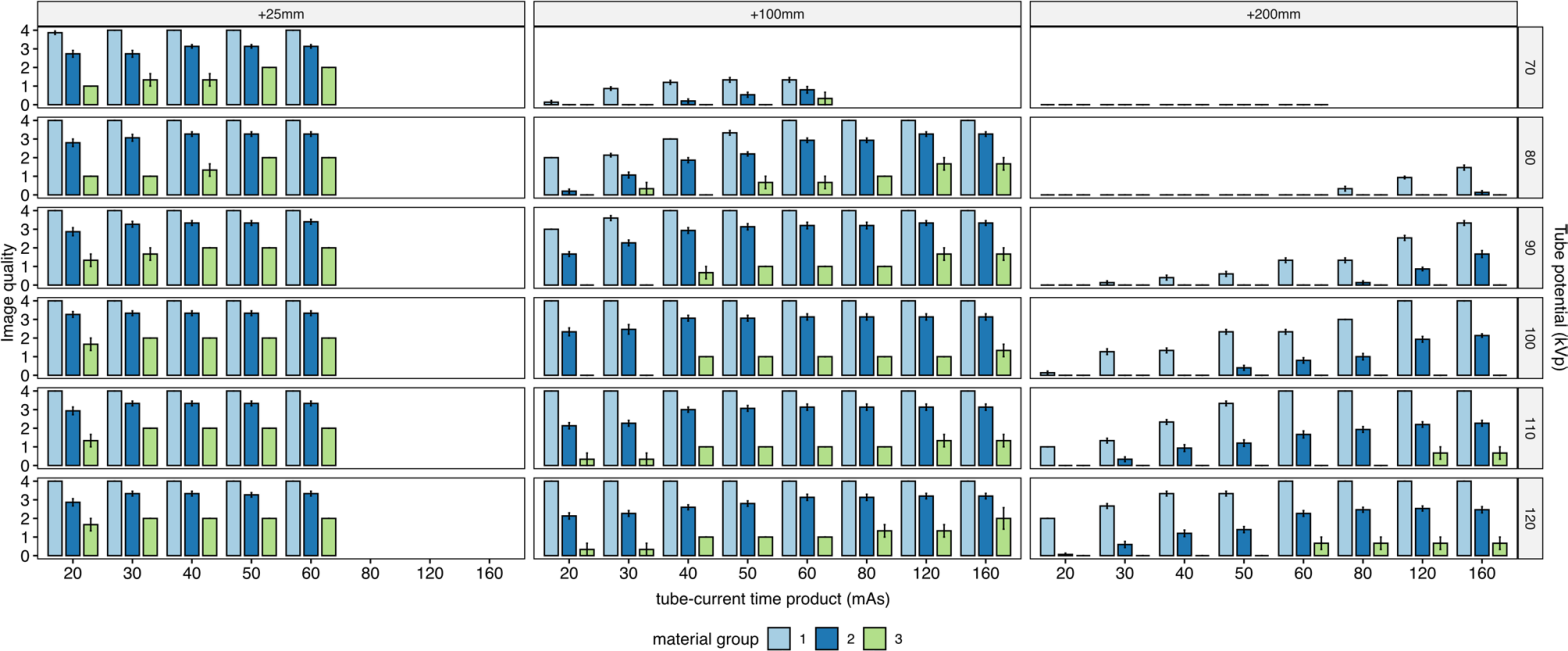
Median image quality scores (+interquartile range) for the different material groups depending on tissue thickness, tube potential and tube-current time product. A score of ≥ 3 (‘good’) is considered sufficient for clinical use.

The device detectability over all readers, devices and scan parameters was significantly different for the different additional tissue thicknesses between the three groups (25 mm (small): median = 3 (good) [IQR 2.5–4.0], 100 mm (medium): median = 3 (good) [IQR 1.5–4], 200 mm (large): median = 0 (not visible) [IQR 0–2]; *p* < 0.05 for all tests). The results are illustrated in Figs. 2 and 3.

The device detectability over all readers, tissue thicknesses and scan parameters was significantly different for the different device groups (group 1: median = 4 (very good) [IQR 2–4], group 2: median = 2.75 (moderate / good) [IQR 0.5–3.0], group 3: median = 1.5 (poor / moderate)[IQR 0–2]; *p* < 0.05 for all tests). The results are illustrated in Figs. 2 and 3.

**Fig. 4:**
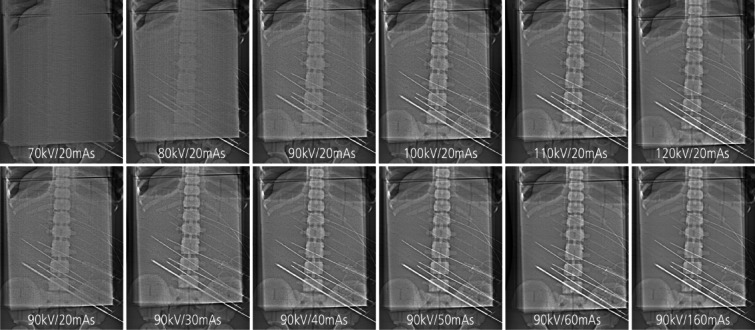
Improvement of detectability of the different devices with increasing tube potential (top row) and increasing tube current (bottom row).

Material detectability over all readers improved with higher doses. This was achieved by increasing either tube current or tube potential (Fig. 4).

#### Cutoff value

As outlined in detail in the methods section, a median visibility of 3 (“good”) or 4 (“very good”) was considered sufficient for clinical routine use.

Devices in group 1 reached a median visibility ≥ 3 for all datasets in the small phantom (CTDI_vol_ ≥ 0.01 mGy), for all datasets ≥ 80 kVp (CTDI_vol_ ≥ 0.02 mGy) in the medium-sized phantom and for all datasets ≥ 100 kVp/ ≥ 80 mAs (CTDI_vol_ ≥ 0.18 mGy) or ≥ 120 kVp/ ≥ 30 mAs (CTDI_vol_ ≥ 0.11 mGy) using the large phantom.

Devices in group 2 reached a median visibility ≥ 3 for all datasets using the small phantom, for datasets ≥ 80 kVp/ ≥ 60 mAs (CTDI_vol_ ≥ 0.06 mGy) or ≥ 90 kVp/ ≥ 40 mAs (CTDI_vol_ ≥ 0.06 mGy) using the medium sized phantom and for all datasets ≥ 120 kVp/ ≥ 120 mAs (CTDI_vol_ ≥ 0.44 mGy) for the large phantom.

The device in group 3 did not reach a median visibility ≥ 3 for all dose levels and phantom sizes.

The results are illustrated in Fig. 3. In summary, the combination of ≥ 80 kVp/ ≥ 60 mAs or ≥ 90 kVp/ ≥ 40 mAs can be regarded as reasonable scan parameters in normal and overweight patients in our CT setup. In obese patients, the scan parameters must be adjusted accordingly (e.g. 120 kVp/120 mAs).

#### Intraclass correlation

A total of 1386 measurements were recorded (25 mm: 6 different tube potentials, 5 different mAs settings, 11 devices / 100 mm and 200 mm: 6 different tube potentials, 8 different mAs settings, 11 devices). The intraclass correlation coefficient (ICC) over all measurements for the three readers were 0.956 [95% CI: 0.952–0.96] for relative agreement (Model: twoway, Type: consistency’, ICC(C,1)) and 0.947 [95% CI: 0.922–0.962] for absolute agreement (Model: twoway, Type: agreement; ICC(A,1)). According to Koo and Li an ICC value above 0.90 can be considered as an excellent agreement^12^. Therefore, the results of the different readers were pooled (median) for the evaluation.

ICC was calculated for the three device groups (1–3). For group 1 (n = 630 / 5 devices) the ICC was 0.974 [0.970–0.978] for relative agreement (ICC(C,1)) and 0.969 [0.957–0.977] for absolute agreement (ICC(A,1)). For group 2 (n = 630 / 5 devices) the ICC was 0.946 [0.937–0.953] for relative agreement (ICC(C,1)) and 0.923 [0.854–0.964] for absolute agreement (ICC(A,1)). For group 3 (n = 126, 1 device) the ICC was 0.924 [0.910–0.936] for relative agreement (ICC(C,1)) and 0.914 [0.886–0.934] for absolute agreement (ICC(A,1)).

#### Dose

The standard settings for the abdominal scout view in clinical routine at the scanner used for this study were 120kVp / 19mAs, equivalent to a calculated CTDI_vol_ of 0.07 mGy as recorded from the CT dose report. When compared to the minimum dose levels necessary to reach the cut-off level as described above, it can be seen, that devices from group 1 and 2 can be reliably visualized in the small and medium sized phantom at dose levels below or comparable to clinical routine scout view acquisition (Table 2). The concept of scout view guided intervention offers potential for dose reduction as a cross-sectional image is replaced with a quasi-projection acquisition.

**Table 2:** Minimum CTDI_vol_ necessary for sufficient image quality (score ≥ 3) depending on material group and phantom size.

Minimum CTDI_vol_	Phantom size		
	Small	Medium	Large
material group 1	≥ 0.01 mGy	≥ 0.02 mGy	≥ 0.18 mGy
material group 2	≥ 0.01 mGy	≥ 0.06 mGy	≥ 0.44 mGy
material group 3	n/a	n/a	n/a
Clinical protocol	0.07 mGy		

Dose from the fluoroscopic image acquisition in the angiography suite was 0.008 mSv (25 mm additional tissue thickness) and 0,01 mSv (10 cm additional tissue thickness) for a 10 cm cranio-caudal collimation, respectively. A comparable acquisition using our scout approach would result in an estimated dose in the range of approximately 0.0015 mSv for the “small” phantom (0.01 mGy*10 cm*0.015 mSv*mGy^−1^*cm^−1^) and 0.003 mSv/0,009 mSv (0.02/0.06 mGy*10 cm*0.015 mSv*mGy^−1^*cm^1^) for the medium sized phantom and material group A / B, respectively.

#### Biopsy—proof-of-concept

**Fig. 5:**
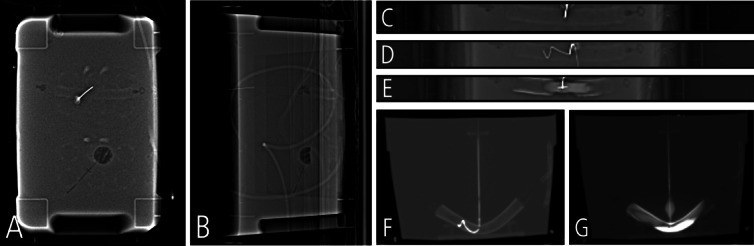
(**A**/**B**): Visualization of the target structure in two planes (scout ap-view/lateral-view) and initial needle placement (**C**): Advancement of the needle into the target structure (scout ap-view). (**D**/**F**): A guidewire is introduced into the target structure (**D**: scout ap-view, **F**: lateral reconstruction of a CT scan for illustrative purposes). **E**/**G**: Iodinated contrast agent is injected into the target structure (**E**: scout ap-view, **F**: lateral reconstruction of a CT scan for illustrative purposes). Scan parameters for the scout were: 100kVp / 50 mAs.

The biopsy was performed as outlined in the Materials & Methods section and is illustrated in Fig. 5. The target in the custom-made phantom was punctured with a needle in a stepwise approach using repetitive (collimated) scout views. The success of the procedure and the potential use in clinical routine was shown by inserting and visualizing a guidewire and by performing a contrast injection in the target structure of the phantom. The depth of the lesion and the success of the puncture was evaluated by tactile feedback (loss of resistance). Additional CT scans were acquired for illustrative purposes.

## Discussion

As described in the introduction, the standard procedure for CT-guided intervention is an iterative approach. Several additional tools are available to facilitate the procedure such as electromagnetic navigation or laser-guidance^13–16^. However, there still is a clinical need for fluoroscopic monitoring of devices in certain indications and the usage of real-time CT scanning (CT fluoroscopy) is limited by a a substantial radiation burden for both, the patient and the interventionalist, despite several technical advances to minimize the exposure^17,18^.

The approach described in this paper utilized the inherent 2D-imaging capabilities of CT scanners to acquire projection-like 2D images. This is applicable in every CT scanner but usually only used to generate the scout views for examination planning. While the image quality of these scout views is inferior when compared with dedicated radiography or fluoroscopy units, our results suggests that it is still sufficient for basic guidance during percutaneous interventions.

The potential advantage of a combined 2D-/3D-approach as described herein is the combination of the static high-resolution cross-sectional image with a potentially dynamic fluoroscopy-like view that allows a real-time control of the needle position. The principle need of combined 2D-/3D-approaches is well documented in the literature with custom made setups and in the portfolio of the manufacturers of imaging equipment with fixed combinations of CT and angio^7,8,19–21^.

There are a couple of shortcomings of the approach described herein that need to be addressed. First, to date, the use of CT scout views for interventional purposes has only been sparsely described in medical literature^22^. However, it is well documented that scout views are more than just simple imaging for planning of CT scans^23^. There are a couple of case reports that describe a potential benefit from including the scout view in routine CT reading as unexpected relevant findings may be identified, thereby underlining a potential diagnostic value of scout views^24,25^. In clinical routine, the position and integrity of central venous catheters is reviewed on scout view prior to their use for high-power flow injection of contrast agents. Furthermore, attenuation measured on scout views is routinely used for automatic exposure controls in CT.

Furthermore, while the scout-view approach does not require additional hardware in or in front of the CT scanner, no implementation of this technique can be found on commercially available scanners yet. There is no software-guidance that facilitates the process of the repetitive fluoro-like acquisition of scout views during an interventional procedure. However, combined 2D-/3D-approaches for biopsy-guidance have already been implemented in advanced angiography and x-ray / fluoroscopy systems or hybrid CT / angiography systems, that combine two different hardware units as described above^19,20^. However, such setups may require patient movement between two modalities whereas the integrated approach does not. As CT is still the most important modality for the guidance of non-vascular interventional procedures, it seems reasonable to upgrade CT with a 2D real-time guidance function. Based on our results we believe that enrichment of the CT scanner software with features, that enable a repetitive acquisition of a collimated scout view in real-time or—at least—in a reasonable time frame will be helpful. The increasing width of CT detectors facilitates the potential use of scout views as larger areas can be covered without table movement.

With respect to patient dose, the CTDI_vol_ value provided by the dose report was 0.07 mGy for the clinical standard ap-scout settings. CTDI_vol_ values given in the literature for CT-fluoroscopy acquisition settings are in the range of 0.50 mGy^18^. A direct comparison of our approach with standard 2D-fluoroscopy remains challenging due to the different physical units, dose parameters and measurement concepts (e.g. phantom vs. air kerma) as well as the associated uncertainties when utilizing different conversion factors. Furthermore, dose in fluoroscopy is highly dependent on the acquisition parameters of the angiography unit that is used for the intervention. However, our indicative comparison with dose values of the same phantom from a standard angiography suite suggests, that the dose of the scout views are of the same order of magnitude.

It is also known that ‘slit-scanning’ techniques show an advantageous dose performance as compared to conventional projection radiography, so it seems reasonable to assume that our approach is dose-efficient^26^. Furthermore, one could argue that the availability of a low-dose scout view might lead to a reduction in the number of control CT scans and thus lead to a dose reduction for the patient. However, this will have to be proven in clinical studies, when scout view technique may be available in clinical scanners.

We only evaluated ap-scout views with respect to image quality, mainly due to our experimental setting using the antropormophic phantom and the additional tissue simulation and device placed upon the phantom. We also used ap-views only in our proof-of-concept study. In a real patient setting, the use of other (arbitrary) viewing angles is usually desirable (e.g. to monitor the depth position of a puncture needle) and—in case of lateral projections—can be readily realized using standard CT equipment. Depending on the body position other scout projections might require a higher dose setting to maintain the same image quality as in ap-views. However, this effect also occurs in standard 2D-fluoroscopy and does not change the overall result of our approach.

Finally, the image quality of scout views of standard CT scanners is usually not optimized to meet diagnostic standards as delivered by dedicated 2D x-ray equipment^23,27,28^. However, we were able to demonstrate that this image quality is still sufficient to guide standard interventional procedures. Since the scout view has traditionally only been used to define the anatomical scan range in CT, it is not optimized for diagnostic image quality, yet. It seems reasonable to expect a significant increase in image quality when the acquisition parameters and post-processing algorithms are specifically adjusted.

In conclusion, we have demonstrated the potential usability of CT scout views as an additional tool in CT-guided intervention in a proof-of-concept study. This approach may facilitate interventional procedure which benefit from a visualization of dynamic events such as contrast injection or guidewire manipulation. Although the image quality will be inferior to a flat panel detector in an angio system, it seems worthwhile to further optimize CT scout view scanning capabilities with respect to fluoroscopy-like imaging, quality of image reconstruction and usability of the user interface.

## Data Availability

The datasets used and/or analysed during the current study are available from the corresponding author on reasonable request.
